# Organic Memristor with Synaptic Plasticity for Neuromorphic Computing Applications

**DOI:** 10.3390/nano13050803

**Published:** 2023-02-22

**Authors:** Jianmin Zeng, Xinhui Chen, Shuzhi Liu, Qilai Chen, Gang Liu

**Affiliations:** 1 School of Electronic Information and Electrical Engineering, Shanghai Jiao Tong University, Shanghai 200240, China; 2 College of Information Engineering, Jinhua Polytechnic, Jinhua 321017, China; 3 AEROSPACE SCIENCE & INDUSTRY SHENZHEN (GROUP) CO., LTD., Shenzhen 518000, China

**Keywords:** artificial synapse, synaptic plasticity, organic memristor, neuromorphic computing, artificial neural networks

## Abstract

Memristors have been considered to be more efficient than traditional Complementary Metal Oxide Semiconductor (CMOS) devices in implementing artificial synapses, which are fundamental yet very critical components of neurons as well as neural networks. Compared with inorganic counterparts, organic memristors have many advantages, including low-cost, easy manufacture, high mechanical flexibility, and biocompatibility, making them applicable in more scenarios. Here, we present an organic memristor based on an ethyl viologen diperchlorate [EV(ClO_4_)]_2_/triphenylamine-containing polymer (BTPA-F) redox system. The device with bilayer structure organic materials as the resistive switching layer (RSL) exhibits memristive behaviors and excellent long-term synaptic plasticity. Additionally, the device’s conductance states can be precisely modulated by consecutively applying voltage pulses between the top and bottom electrodes. A three-layer perception neural network with in situ computing enabled was then constructed utilizing the proposed memristor and trained on the basis of the device’s synaptic plasticity characteristics and conductance modulation rules. Recognition accuracies of 97.3% and 90% were achieved, respectively, for the raw and 20% noisy handwritten digits images from the Modified National Institute of Standards and Technology (MNIST) dataset, demonstrating the feasibility and applicability of implementing neuromorphic computing applications utilizing the proposed organic memristor.

## 1. Introduction

In the big data era, huge volumes of data with much irregularity are rapidly generated every day, which is becoming a big challenge for information processing systems [1,2,3]. Recently, emerging neuromorphic computing methodologies, including artificial neural networks (ANNs), have attracted much attention due to their abilities to imitate the functions of synapses and neurons of the human brain and to deal with tricky tasks involving big data [4,5,6]. Numerous studies have also demonstrated their successful application in a wide range of fields in relationship with big data, such as computer vision (CV), pattern recognition, and natural language processing (NLP) [7,8,9]. However, the existing neuromorphic computing hardware platforms, including the General-Purpose Graphics Processing Unit (GPGPU) and application-specific accelerators such as Google’s Tensor Processing Unit (TPU) [10], are mainly designed on the basis of the traditional von Neumann architecture, where the computation and memory units are physically separated from each other, inevitably incurring frequent data movement back and forth between them. Consequently, this leads to the memory wall problem and brings about a performance bottleneck and low-energy efficiency to the system [11,12]. The shortcomings are exposed more obviously especially when the von-Neumann-architecture-based systems are used to deal with data-intensive applications such as neuromorphic computing. As in the human brain, synapses are the fundamental and key elements of a neuromorphic system. The synapses in existing neuromorphic systems are usually realized using CMOS devices, hindering the design of synapses and neurons with a higher density in neuromorphic systems. Therefore, it is quite essential to explore novel devices beyond von Neumann computing paradigms to design more performance- and energy-efficient synapses, neurons, as well as neuromorphic computing systems.

Recently, memristors have been widely used to design artificial synapses and neurons, owing to their numerous advantages such as such as nonvolatility, high density, low-power consumption, and CMOS compatibility [13,14,15,16,17,18]. The resistive switching and multiconductance state properties allow the memristor-based synapses and neurons to do in situ computing in an analogue fashion with the help of the Ohm’s Law and Kirchhoff’s Current Law (KCL), inherently fusing the functions of memory and computation into the identical devices [19]. As a result, the frequent data movement problem can be addressed, improving both the performance and energy efficiency of the system. Additionally, the nonvolatility of memristors enables the stored synaptic weights to be kept unchanged even when the system is powered off, which can further lower the overhead during a system initiation. Furthermore, when stimuli of appropriate amplitudes and widths are applied, biological synaptic behaviors such as spike-timing-dependent plasticity (STDP), potentiation, and depression can be observed on memristors [20]. Afterwards, a neuromorphic computing system beyond the von Neumann paradigm could be established where memristors are utilized to construct artificial electronic synapses and neurons. Therefore, memristors are considered to be one of the most promising next-generation neuromorphic devices. Numerous reports [20,21,22,23,24,25,26,27,28,29,30] have broadly investigated and demonstrated the applicability of memristors in such fields. However, most of them employed memristors based on inorganic materials. For instance, memristors based on metal oxide materials such as HfO_x_ or ZnO were used to realize artificial synapses in [20,21,22], while sulfide materials such as Ag_2_S or Cu_2_S were used in [23,24]. Additionally, other works [27,28,29] also employed memristors based on 2D materials to realize artificial synapses and neuromorphic computing systems. Compared with inorganic counterparts, memristors based on organic materials have many more strengths, including low cost, easy manufacture, high-mechanical flexibility, biocompatibility, and more importantly, tunable electronic properties [19,31,32]. This allows them to be applied in scenarios such as wearable devices or even skin-implantable systems.

In this work, a two-terminal organic memristor using ethyl viologen diperchlorate [EV(ClO_4_)]_2_/triphenylamine-containing polymer (BTPA-F) as the resistive switching layer (RSL) is presented. The bilayer-structured RSL between two metal electrodes exhibits memristive behavior and excellent long-term synaptic plasticity, which is of great importance for artificial synapses and neurons. Additionally, the conductance states can be precisely modulated by applying appropriate voltage pulses, making it possible to design multilevel-weight synapses using the device. A multilayer perception (MLP) neural network [33] was designed and implemented using the EV(ClO_4_)_2_/BTPA-F-based memristor to examine the feasibility of implementing neuromorphic computing systems employing the proposed device. By taking advantage of the nonvolatile and tunable conductance of the device and the KCL, the simulated hardware network is capable of storing synaptic weights and doing neuromorphic computing on identical devices, which can significantly improve the performance and lower the power of a neuromorphic computing system. The synaptic weights of the network were trained and modulated on the basis of the memristor’s synaptic plasticity characteristics and conductance modulation rules. Recognition accuracies of 97.3% and 90% were achieved, respectively, for the raw and 20% noisy handwritten digits images from the Modified National Institute of Standards and Technology (MNIST) dataset [34]. Thus, the EV(ClO_4_)_2_/BTPA-F-based organic memristor is a promising candidate for neuromorphic computing applications.

## 2. Materials and Methods

All chemical reagents were purchased from Aldrich (Shanghai, China) without further purification. The ^1^H nuclear magnetic resonance (^1^H NMR) spectra were conducted at 400 MHz on a Bruker 400 AVANCE III spectrometer (Bruker, Billerica, MA, USA) with deuterated chloroform as solvent and tetramethylsilane (TMS) as a standard chemical shift of zero. Weight-average (M_w_) and number-average (M_n_) molecular weights were recorded by a waters 2690 gel permeation chromatography (Tosoh Corporation, Tokyo, Japan) unitizing polystyrene standards and elution with tetrahydrofuran solvent (THF, 1 mL/min). The UV-Visible absorption spectra characterization was measured in 10 μM solution on a Shimadzu UV-2450 spectrophotometer (Shimadzu, Kyoto, Japan). Steady-state fluorescence spectra of the polymer devices were recorded on an Andor SR303i-A/DU420A-BVF spectrofluorometer (Oxford Instruments, Abingdon, Oxfordshire, UK). Cyclic voltammetry (CH Instruments Inc., Austin, TX, USA) measurements were recorded in the tetrabutylammonium perchlorate (*n*-Bu_4_NClO_4_) solution of acetonitrile (0.1 M) under an argon atmosphere utilizing platinum gauze and Ag/AgCl as the counter and reference electrodes, respectively, in which a typical scan rate of 50 mV/s was used during the CV measurements. The cross-sectional images of the BTPA-F nanofilm as well as the BTPA-F/EV(ClO_4_)_2_ bilayer structure was conducted on a Hitachi S-4800 field-emission scanning electron microscope (Hitachi, Kyoto, Japan). The I-V sweeps were measured by Agilent B1500 semiconductor analysis system (Agilent Technologies Inc., Palo Alto, CA, USA) at room temperature. The deposition of the top electrode utilized the magnetron sputtering system at the Ar atmosphere.

**Figure 1:**
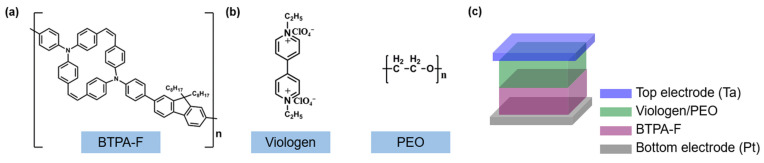
Device structure of the presented organic memristor. (**a**,**b**) The chemical structure of the BTPA-F and EV(ClO_4_)_2_ and PEO as anion acceptor, donor, and film-forming assistant, respectively. (**c**) Schematic illustration of the memristor device with Ta and Pt as the top and bottom electrodes, and the bilayer-structured EV(ClO_4_)_2_/BFPA-F as the resistive switching layer.

Further, we utilized the electron-rich polymer of BTPA-F that can accept the anion of electron deficiency (Figure 1a) and the electron deficiency molecule viologen as an anion acceptor (Figure 1b) to form the push and pull anion effect. Considering the solution ability of the viologen organic salt dissolving in the organic solvent due to its high molecule polarity, we dispersed them in the PEO polymer to generate the even viologen: PEO nanofilm. Under the outside field, the perchlorate in viologen can be stimulated to migrate to the BTPA-F polymer coordinating with the amino, thus forming the variation of the electronic transportation. Reversely, changing the polarity of the outside field can also pull the perchlorate back to the viologen to fulfill an entire cycle. Benefiting from the promising anion migration mechanism from the molecule design, we fabricated the EV(ClO_4_)_2_/BFPA-F bilayer memristive device to conduct the electrical measurement. The device fabrication method is introduced, as below, sequentially: (1) Washing the Pt-coated Si/SiO_2_ substrate with the water, alcohol, and acetone for 30 min; (2) Spin coating the 30 μL solution of the BTPA-F (5 mg/mL) with 3000 rpm for 60 s before drying at 60 °C for 6 h; (3) Spin coating the 50 μL solution of the viologen (3 mg/mL) with 3500 rpm for 45 s before drying at 60 °C for 6 h; (4) Top electrode Ta disposition by the magnetron sputtering system (Figure 1c).

## 3. Results and Discussion

### 3.1. Electrical Characteristics and Long-Term Synaptic Plasticity

**Figure 2:**
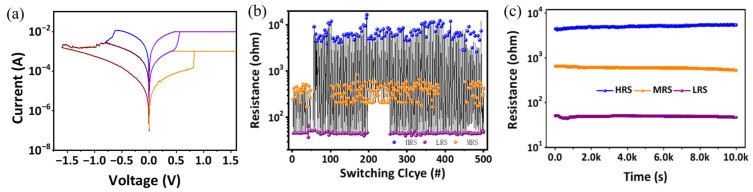
Electrical parameters of the memristor. (**a**) Multistate I-V characteristic curve of memristor; (**b**) The device endurance and (**c**) retention at room temperature.

**Figure 3:**
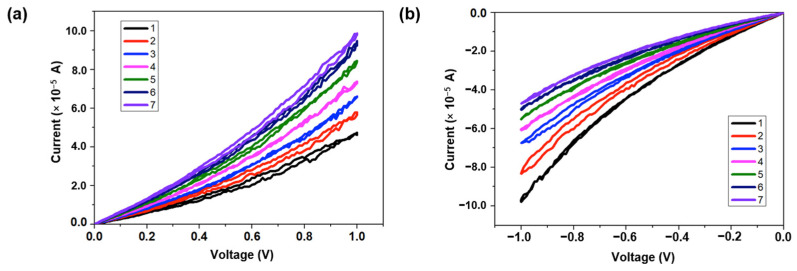
Current–voltage characteristics of the Ta/EV(ClO_4_)_2_/BFPA-F/Pt memristor when subjected to (**a**) consecutive positive voltage sweeps (0 V → 1 V → 0 V) and (**b**) consecutive negative voltage sweeps (0 V → −1 V → 0 V). Small voltages of 0.2 V and −0.2 V are used to read the positive and negative current responses, respectively.

The EV(ClO4)_2_/BTPA-F RSL exhibits memristive behavior when sandwiched between the top electrode tantalum and the bottom electrode platinum. Compared with the related literature [35,36,37,38], the devices prepared with the dielectric materials in this manuscript have more stable linear conductance states, as well as better endurance and biocompatibility. The current–voltage curve of the device is shown in Figure 2a. During the set process, the window displayed by the high- and low-resistance-state transitions indicates that there is a large switching ratio (>10). During the reset process, there are many slowly changing stable and controllable resistance states rather than instantaneous flipping. Taking the ternary conductance as a typical value, the electrical parameters of the system test the device at room temperature. Experimental results show that the conductance state can be repeatedly programmed and accessed within 500 cycles (Figure 2b) and remains for at least 10^4^ s (Figure 2c). It was found that, by means of ion transport and compensatory doping, electrons can be removed from the main chain of triphenylamine polymers through redox to generate holes which can not only increase the concentration of mobile carriers but also generate a new polaron energy level in the original energy gap. Thus, the carrier mobility can be further adjusted by taking advantage of the change of the energy level between the adjacent groups. Consequently, the conductance of the bilayer-structured RSL can be precisely modulated. Experiments were conducted to observe the tuning process of the memristive behavior. As indicated in Figure 3a, the conductance of the device will gradually increase (from 0.04 mS to 0.1 mS) when consecutive positive voltage sweeps of 0 V → 1 V → 0 V are applied to the top and bottom electrodes of the device. Correspondingly, the conductance of the device will decrease (from 0.05 mS to 0.1 mS) when consecutive negative voltage sweeps of 0 V → −1 V → 0 V are applied to the device, as shown in Figure 3b. Utilizing the nonvolatility of the device, applying the same positive or negative scanning voltage to it sequentially seven times, the conductance value will show seven continuously increasing or decreasing conductance values. The curves of different colors in the figure represent different memristive states, and the variation range of the resistive state shows excellent symmetry. Different from those bistable memristors with abrupt changing conductance [39,40], our device shows a slower and smoother conductance tuning trend, which is more useful in artificial electronic synaptic applications [31].

**Figure 4:**
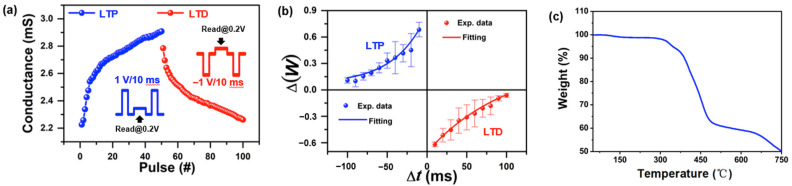
Long-term synaptic plasticity of the Ta/EV(ClO_4_)_2_/BTPA-F/Pt memristor. (**a**) Long-term potentiation and depression characteristics by applying rapid and repetitive positive and negative voltage pulses to the device. A small voltages of ± 0.2 V are used to read the current responses and calculating the corresponding conductance. (**b**) Schematic illustration of the spike-timing-dependent plasticity properties of the device. (**c**) Synaptic weight retention performance of the device in response to temperature change.

The conductance value is positive and the weight has positive and negative values. Here, we add the maximum value (Gmax) of the device to the minimum value (Gmin) and then divide the sum by two. Use this value as the critical point. If the conductance value of the device is greater than this value, it will be a positive weight, otherwise it will be negative. When the activity between the presynaptic neuron and postsynaptic neuron increases or decreases, the synaptic connection will be strengthened or weakened. The change in the strength of the synaptic connection is defined as synaptic plasticity [41]. As one of the basic elements of synaptic plasticity, long-term plasticity indicates long-lasting changes in synaptic weight and is believed to be related to the learning and memory mechanisms in the human brain [42]. The phenomenon of the long-lasting or permanent increase in synaptic weight is referred to as long-term potentiation (LTP). By contrast, the phenomenon of long-lasting or permanent decrease in synaptic weight is referred to as long-term depression (LTD) [43]. LTP and LTD can be used as the basic rules of synaptic weight renewing and modulating in neuromorphic computing systems. The Ta/EV(ClO_4_)_2_/BFPA-F/Pt memristor can be used as electrical synapse with synaptic plasticity, where the top electrode tantalum acts as the presynaptic neuron while the bottom electrode platinum acts as the postsynaptic neuron. Figure 4a depicts the conductance response of our device on applying consecutive positive or negative voltage pulses, which demonstrates the LTP and LTD properties of the memristor. To begin with, 50 consecutive positive voltage pulses with the amplitude of 1 V, duration of 10 ms, and period of 2 s are applied to the Ta/EV(ClO_4_)_2_/BFPA-F/Pt memristor. Subsequently, 50 consecutive negative voltage pulses with the identical amplitude, duration, and period were immediately applied to the device. The positive and negative voltage pulse stimuli caused the occurrence LTP and LTD, as indicated by the blue and red curves, respectively.

Additionally, the LTP and LTD can also be charactered by the spike-timing-dependent plasticity (STDP) [44,45]. STDP is a temporally asymmetric Hebbian learning rule induced by tight temporal correlations between presynaptic and postsynaptic neuronal spikes through which the connection strength between neurons can be modulated [46,47]. Figure 4b demonstrates the STPD properties of the Ta/EV(ClO_4_)_2_/BTPA-F/Pt memristor through the schematic illustration of the anti-STDP window [48,49]. ΔW denotes the synaptic weight change in the device and can be calculated by the following equation [50]

(1) $$\Delta W=\frac{I_{post}-I_{pre}}{I_{pre}}$$

where $I_{post}$ and $I_{pre}$ denote the current of presynaptic and postsynaptic spikes and $\Delta t\;(t_{post}-t_{pre})$ denotes the time interval between the post- and presynaptic spikes. When the postsynaptic spike arrives before the presynaptic (Δt<0), the synaptic weight change (ΔW) will be positive, wherein the value of synaptic weight will increase gradually, indicating an LTP process. On the contrary (Δt>0), the synaptic weight change will be negative wherein the value of synaptic weight will decrease gradually, indicating an LTD process.

The synaptic weight retention performance of our device in response to temperature change is also examined in this work. The result shown in Figure 4c demonstrates that our device can tolerate a wide range of temperature without obvious synaptic weight loss, which allows the device to be applied in a wide range of temperature environment.

### 3.2. Neuromorphic Network Implementation

**Figure 5:**
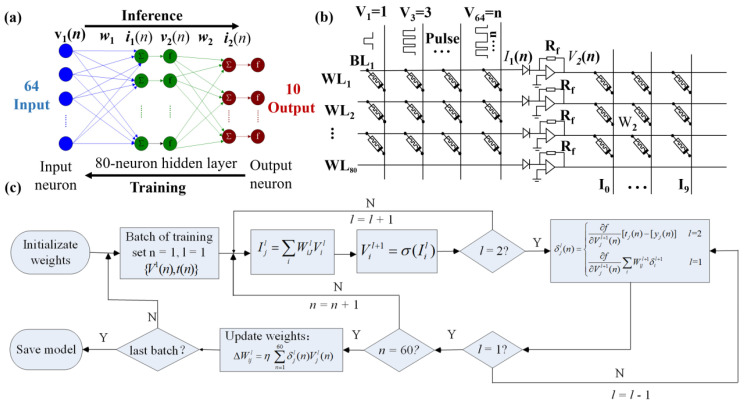
A demonstration of implementation of neuromorphic computing utilizing the proposed device. (**a**) Custom three-layer MLP containing 64 input neurons, 80 hidden neurons, and 10 output neurons. (**b**) Dedicated crossbar array based on the presented memristor for implementing the MLP hardware. (**c**) The training process flowchart of 60,000 images, where l, i, j, and k belong to the range of [1,2], [1,64], [1,80], and [1,10], respectively. These indexes imply the sequence number of the input pixels, hidden neurons, and output results, respectively.

The biological presynapse and postsynapse can be respectively mapped to the top and bottom electrodes of the memristor, and the conductance value corresponds to the synaptic weight. Applying pulse voltage on the memristive device can be used to replace the nerve stimulation signal of neurons. The characteristics of nerve stimulation signals corresponding to different synaptic functions can be simulated by changing the shape, frequency, duration, and other parameters of the pulse voltage. As shown in Figure 5a, a three-layer MLP neural network was designed and implemented utilizing the Ta/EV(ClO_4_)_2_/BTPA-F/Pt memristor for the purpose of demonstration of the feasibility of our device in implementing neuromorphic computing systems. The supervised learning based on the backpropagation (BP) algorithm was employed to train the network using 60,000 images from the MNIST database, a standard benchmark widely used to gauge machine learning algorithms. The grayscale of the image is represented by the conductance value. According to the corresponding relationship between the pulse and the memristive state, the grayscale of the image in the database is mapped to the number of spike pulses that need to be applied. Each input image was scaled to 8 pixels by 8 pixels to match up the size of our custom network. Through cropping and bicubic interpolation downsampling methods, the effective information of the image is preserved under the condition of adapting the input quantity of the network. It is worth mentioning that the more integrated memristive network has more input features, thus achieving higher resolution image recognition. A total of 64 input neurons of the network corresponded to the total amount of pixels of one image, while 10 output neurons corresponded to 10 handwritten Roman numerals. The weights were updated during the learning process based on the experimental data sampled by testing on the Ta/EV(ClO_4_)_2_/BTPA-F/Pt synaptic memristor according to the LTP and LTD modulation rules. A dedicated crossbar array based on the presented memristor was then designed to simulate the custom neural network, as illustrated in Figure 5b. The top electrodes of the devices on an identical row were connected to a word-line (WL) while the bottom electrodes of the devices on an identical column were connected to a bit-line (BL). The top and bottom electrodes of each individual memristor device mimicked the pre- and postsynaptic neuron, respectively, while the bilayer-structured RSL of the device acted as the synapse. The custom MLP neural network consist of 5920 (80 rows × 74 rows) artificial synapses, each of which was initialized to the minimum conductance of the presented. The training dataset from MNIST was used in the training duration, with a mini-batch size of 60. As illustrated in Figure 5c, the network training was composed of two stages: feedforward inference and feedback weight update. The synaptic weight of each synapse was kept unchanged and used in each feedforward inference iteration and updated in each backward iteration by applying voltage pulses according to the LTP and LTD modulation rules of the device. The feedforward inference was performed layer by layer sequentially, as was the backward weight update. The input voltage vector for the first layer was a feature vector from the dataset, while the input vector for the subsequent layer was the output vector of the previous layer. The analogue weighted sum can be performed along bit-lines according to the Ohm’s law and Kirchhoff’s law [51,52], demonstrating that the in situ computing is enabled in the memristor array. The total current of each bit-line was the summation of the currents through each device in the same column, while each current was the product of the conductance and the corresponding voltage across the memristor. The input signal of hidden neurons can be derived from the Equation (2):

(2) $$I_{j}^{l}=\sum_{i=1}^{64}W_{ij}^{l}V_{i}^{l}$$

where $V_{i}^{l}$ denotes the input voltage vector applied to the top electrodes of the synaptic devices, $I_{j}^{l}$ denotes the readout current vector from the bottom electrodes of the synaptic devices, while $W_{ij}^{l}$ denotes the weight matrix of layer l. Then, the current results were activated by a nonlinear sigmoid transfer function. The activated result of a hidden layer was transferred to the output neurons. The inference result was calculated by Equation (3):

(3) $$V_{i}^{l+1}=\sigma (I_{i}^{l})=\left\{ \begin{array}{ll} cI_{i}^{l}, & I_{i}^{l}>0\\ 0, & I_{i}^{l}\leq 0 \end{array}\right.$$

where *c* is 800 V/A, which is a scaling factor matching the voltage range of the device. The resulting voltage elements exceeding 0.8 V were clipped to avoid changing the memristor state. $V^{2}$ and $V^{3}$ are the output signals of the second and third layers of the network, respectively. The forward propagation process ends at this point.

During the BP process, delta weights are calculated and transferred to modify the synaptic weights with the driving circuit, as the Equations (4) and (5) show:

(4) $$\delta_{j}^{l}(n)=\left\{ \begin{array}{ll} \frac{\partial f}{\partial v_{j}^{l+1}(n)}[t_{j}(n)-y_{j}(n)] & l=2\\ \frac{\partial f}{\partial v_{j}^{l+1}(n)}\sum_{i}W_{ij}^{l+1}\delta_{i}^{l+1} & l=1 \end{array}\right.$$

where $y_{j}(n)$ and $t_{j}(n)$ represent the input feature vector and the target output vector (label), respectively; $v_{j}^{l+1}$ and *f* denotes the output voltage vector of the postsynaptic electrode and the activation function, respectively.

(5) $$\Delta W_{ij}^{l}=\eta \sum_{n=1}^{60}\delta_{j}^{l}(n)V_{i}^{l}(n)$$

where η is the learning rate and $\delta_{j}^{l}$ is the calculated error between the real output and the corresponding target value during the training process. When the feedback was transferred to the weights of the first layer, an epoch finished.

**Figure 6:**
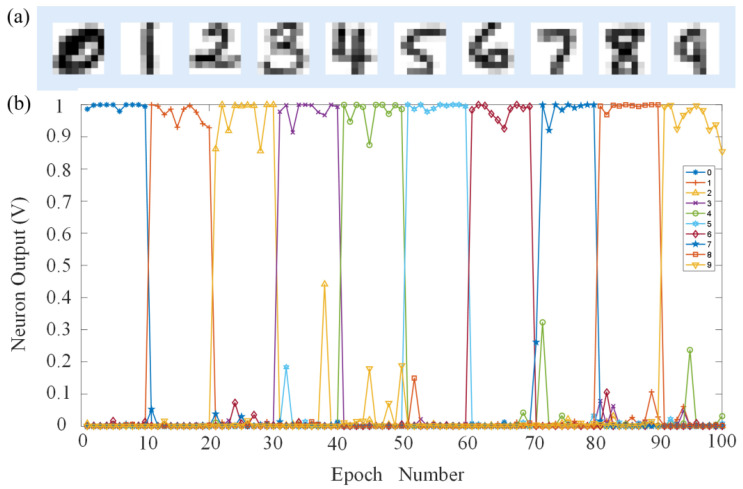
Pattern classification utilizing the dedicated memristor crossbar. (**a**) Handwritten digits from the MNIST database. (**b**) The evolution of output signals averaged over all patterns of a specific class.

**Figure 7:**
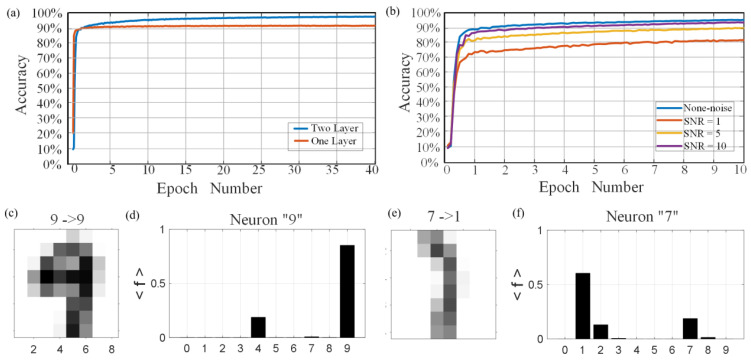
Training results. (**a**) Recognition accuracy in response to neural network structure with different layer numbers. (**b**) The recognition accuracy of the neural network with the same structure under different signal-to-noise ratios. (**c**,**d**) Correctly classified digit “9” and (**e**,**f**) misclassified digit “7”.

The MNIST database contains a total of 70,000 handwritten digital pictures, of which 60,000 are used to train the neural network and the remaining 10,000 are used to test and validation the result of network accuracy. There are two training methods: online and offline. Online training is real-time training and testing in the hardware circuit. Offline training is training to obtain weights first and then adjusting the memristor conductance value. Online training requires more calculations and time and is prone to overfitting; therefore, we adopted offline training. The training database was used to train the custom MLP neural network. After being trained for 40 epochs, the recognition performance of the network implemented using the presented memristor was examined using the testing database (Figure 6a) from the same database. After a feedforward inference process, the maximum value of the output neuron was taken as the inference result. As a result, a recognition accuracy of 97.3% was achieved. Figure 6b shows the inference results for the ten digits from “0” to “9”, indicating that the neural network based on the presented memristor exhibits excellent recognition performance. Figure 7a shows the recognition accuracy in response to neural network structure with different layer numbers. With the increase of training epochs, the recognition accuracy of the neural network with two hidden layers is significantly higher than that with one layer. The performance of the custom network under different Signal-to-Noise Ratio (SNR) was then tested in this work (Figure 7b). We used MATLAB (2020a, Natick, MA, USA) to randomly generate noisy matrices with different signal-to-noise ratios and superimposed them on the original image matrix, where the noise amplitude obeyed Gaussian distribution. The recognition accuracies of 93.7%, 90%, and 81.3% were achieved under an SNR of 10, 5, and 1, respectively, indicating that the custom neural network implemented using the presented memristor can tolerant a relatively high noise contamination. Figure 7c–f show two cases indicating that noise contamination may bring about accuracy drop for the recognition task.

## 4. Conclusions

In summary, a redox system consisting of ethyl viologen diperchlorate ([EV(ClO_4_)]_2_) and triphenylamine-containing polymer (BTPA-F) was fabricated and used as the RSL of the organic memristor. When sandwiched between two metal electrodes, the bilayer-structured RSL exhibits memristive behaviors and excellent long-term synaptic plasticity. We evaluated the electrical characteristics and memristive behaviors of the memristor by applying consecutive positive and negative voltage sweeps for conductance setting and resetting, and small voltages of ± 0.2 V for current reading. The long-term potentiation and long-term depression properties together with the weight modulation rules were then investigated to verify the long-term plasticity of the device. At last, a three-layer artificial neural network was designed and implemented by customizing a synaptic crossbar array employing the presented memristor as the synapses. The experimental results demonstrate the feasibility and applicable of our device in implementing neuromorphic computing systems.

## Data Availability

Not applicable.
